# Prognostic Significance of Integrin Subunit Alpha 2 (ITGA2) and Role of Mechanical Cues in Resistance to Gemcitabine in Pancreatic Ductal Adenocarcinoma (PDAC)

**DOI:** 10.3390/cancers15030628

**Published:** 2023-01-19

**Authors:** Alessandro Gregori, Cecilia Bergonzini, Mjriam Capula, Giulia Mantini, Fatemeh Khojasteh-Leylakoohi, Annalisa Comandatore, Ghazaleh Khalili-Tanha, Alireza Khooei, Luca Morelli, Amir Avan, Erik H. Danen, Thomas Schmidt, Elisa Giovannetti

**Affiliations:** 1 Department of Medical Oncology, Cancer Center Amsterdam, Amsterdam UMC, Vrije Universiteit, 1081 HV Amsterdam, The Netherlands; 2 Department of Cancer Biology and Immunology, Cancer Center Amsterdam, 1081 HV Amsterdam, The Netherlands; 3 Leiden Academic Center for Drug Research, Leiden University, 2333 CC Leiden, The Netherlands; 4 Institute of Life Sciences, Sant’Anna School of Advanced Studies, 56127 Pisa, Italy; 5 Cancer Pharmacology Lab, Fondazione Pisana per La Scienza, 56017 San Giuliano, Italy; 6 Metabolic Syndrome Research Center, Mashhad University of Medical Sciences, Mashhad 91886-17871, Iran; 7 General Surgery Unit, Department of Translational Research and New Technologies in Medicine and Surgery, University of Pisa, 56100 Pisa, Italy; 8 Department of Pathology, Faculty of Medicine, Mashhad University of Medical Sciences, Mashhad 91886-17871, Iran; 9 Medical Genetics Research Center, Faculty of Medicine, Mashhad University of Medical Sciences, Mashhad 91886-17871, Iran; 10 Physics of Life Processes, Huygens-Kamerlingh Onnes Laboratory, Leiden University, 2333 CA Leiden, The Netherlands

**Keywords:** pancreatic cancer, gemcitabine resistance, prognostic biomarkers, integrins, mechanobiology, stiffness

## Abstract

**Simple Summary:**

Pancreatic ductal adenocarcinoma (PDAC) is an aggressive and chemoresistant cancer, with a poor overall survival. The stiff stroma surrounding PDAC is known to be involved in chemoresistance via mechanical cues, although the mechanisms behind it are poorly understood. Here, we investigated whether integrin alpha 2 (ITGA2), a mechanical sensor of stiffness, correlated with poor prognosis and whether matrix stiffness could trigger chemoresistance to gemcitabine. By assessing two cohorts of patients, we observed a poorer prognosis for the high ITGA2 expression group. An increase in matrix stiffness prompted cancer cells to express more ITGA2 and become chemoresistant. ITGA2 might therefore be an interesting therapeutic target to overcome resistance to gemcitabine.

**Abstract:**

Introduction: PDAC is an extremely aggressive tumor with a poor prognosis and remarkable therapeutic resistance. The dense extracellular matrix (ECM) which characterizes PDAC progression is considered a fundamental determinant of chemoresistance, with major contributions from mechanical factors. This study combined biomechanical and pharmacological approaches to evaluate the role of the cell-adhesion molecule ITGA2, a key regulator of ECM, in PDAC resistance to gemcitabine. Methods: The prognostic value of ITGA2 was analysed in publicly available databases and tissue-microarrays of two cohorts of radically resected and metastatic patients treated with gemcitabine. PANC-1 and its gemcitabine-resistant clone (PANC-1R) were analysed by RNA-sequencing and label-free proteomics. The role of ITGA2 in migration, proliferation, and apoptosis was investigated using hydrogel-coated wells, siRNA-mediated knockdown and overexpression, while collagen-embedded spheroids assessed invasion and ECM remodeling. Results: High ITGA2 expression correlated with shorter progression-free and overall survival, supporting its impact on prognosis and the lack of efficacy of gemcitabine treatment. These findings were corroborated by transcriptomic and proteomic analyses showing that ITGA2 was upregulated in the PANC-1R clone. The aggressive behavior of these cells was significantly reduced by ITGA2 silencing both in vitro and in vivo, while PANC-1 cells growing under conditions resembling PDAC stiffness acquired resistance to gemcitabine, associated to increased ITGA2 expression. Collagen-embedded spheroids of PANC-1R showed a significant matrix remodeling and spreading potential via increased expression of CXCR4 and MMP2. Additionally, overexpression of ITGA2 in MiaPaCa-2 cells triggered gemcitabine resistance and increased proliferation, both in vitro and in vivo, associated to upregulation of phospho-AKT. Conclusions: ITGA2 emerged as a new prognostic factor, highlighting the relevance of stroma mechanical properties as potential therapeutic targets to counteract gemcitabine resistance in PDAC.

## 1. Introduction

Pancreatic cancer is one of the most aggressive types of cancer, with a 5-year survival rate of only 10% in United States [1] and a mortality rate which almost equals the incidence, as assessed by the Global Cancer Statistics in 2020 [2]. Pancreatic ductal adenocarcinoma (PDAC) is the most prevalent form of pancreatic cancer. Concurring factors for the poor prognosis are late and difficult diagnosis, lack of specific biomarkers, early metastatic spread and limited response or resistance to currently available therapies [3]. Up to now, the only curative option for PDAC is surgery, which is, however, limited to a small percentage of patients. Patients not eligible for surgery are treated with different chemotherapy regimens [4]. FOLFIRINOX (5-fluorouracil, leucovorin, irinotecan, and oxaliplatin) and gemcitabine/nab-paclitaxel are the two current standards of care first-line treatment regimens for advanced PDAC, while gemcitabine monotherapy is still used to treat unfit patients [5].

The efficacy of available treatments is often impaired by chemoresistance, which can be innate or acquired. Many factors have been proposed that concur to PDAC chemoresistance. These can be categorized in cell-intrinsic mechanisms, including altered expression of drug transporters or activation of survival signalling pathways, and extrinsic mechanisms, such as the influence that the tumor microenvironment can have on drug uptake, or on gene expression patterns [6]. However, the increased knowledge of PDAC biology, including the identification of specific subtypes, has not yet been translated to the development of novel effective therapies to restore chemosensitivity in refractory patients [7]. Therefore, there is an urgent need to discover additional mechanisms and targets to tackle chemoresistance in PDAC.

During PDAC progression, hallmark genetic alterations in *KRAS*, *TP53*, *p16* and *SMAD4* are accompanied by a massive desmoplastic reaction, in which tumor-surrounding stromal cells are activated by cancer cells to deposit an extracellular matrix (ECM), which leads to a structural modification of pancreatic tissue [8]. Common features of this desmoplastic reaction include the unique composition of immune cells creating a strongly immunosuppressive environment and the occurrence of mechanical alterations which can deform and even collapse blood and lymphatic vessels, limiting the flow of nutrients, oxygen, and therapeutics [9,10,11]. Of note, such drastic changes of the microenvironment during PDAC progression in turn influence the behaviour of tumor cells. Indeed, the increased stiffness and mechanical feature of the desmoplastic reaction has been involved in increased invasive potential, as well as in chemoresistance of PDAC cells [12,13].

Integrins are heterodimeric transmembrane glycoproteins that provide the main adhesion site for cells to the microenvironment, and mediate the adhesion to surrounding cells, allowing them to mechanically communicate with each other. The dynamic nature of integrin receptors allows cells to sense and adjust to the mechanical and chemical cues from the environment, and vice versa, to apply forces on the surrounding environment [14]. Dysregulation of their physiological expression and activity has been associated with uncontrolled proliferation, invasion, increase in cancer cell stemness and therapy resistance, in multiple tumors [15,16]. High expression levels of the Integrin α2 (ITGA2) subunit have been associated with aggressiveness, increased invasion and poor prognosis in several solid cancers [17,18,19,20]. In particular, ITGA2 has recently emerged as a potential prognostic biomarker and therapeutic target in PDAC [21,22]. However, the impact of ITGA2 on PDAC chemoresistance has not yet been elucidated.

In this study, we aimed to explore the prognostic significance of ITGA2 in addition to the role of mechanical cues in resistance to gemcitabine in PDAC. We firstly demonstrated the prognostic value of ITGA2 in PDAC using the GEPIA database and two cohorts of radically resected and metastatic PDAC patients treated with gemcitabine. We then investigated the role of ITGA2 in gemcitabine resistance by measuring its expression in the PANC-1 cell line and its gemcitabine-resistant clone PANC-1R with label-free proteomics and RNA-sequencing. Surprisingly, we found an increased expression of ITGA2 in the resistant cells. Furthermore, by performing immunocytochemistry on PDAC cells grown on plastic or on hydrogel-coated plates with stiffnesses resembling that of healthy tissue and PDAC environments, we found increased expression of ITGA2 on the stiffer hydrogels. We further explored the effect of matrix stiffness on gemcitabine sensitivity in PDAC cells, and on 3D invasion from tumor spheroids in collagen matrices with varying stiffness. We next explored the role of ITGA2 in invasion, proliferation, and apoptosis using siRNA-mediated knockdown on hydrogel-coated plates with different stiffness. Finally, we overexpressed ITGA2 in MiaPaCa-2 cells and assessed gemcitabine sensitivity, as well as the phosphorylation of AKT, which has been previously associated to gemcitabine chemoresistance [23,24]. Modulation of ITGA2 expression was also evaluated in vivo for further pre-clinical validation. Remarkably, we found that ITGA2 expression modulates cells’ sensitivity to gemcitabine, supporting future studies on personalized treatments targeting ITGA2 in PDAC.

## 2. Materials and Methods

### 2.1. Cells and Culture Conditions

For this study, the pancreatic cancer cell line PANC-1 and a resistant variant to gemcitabine (PANC-1R) were kindly supplied by the Institute for Surgical Research, Philipps-University of Marburg, Marburg, Germany [25]. A PANC-1R clone was selected by continuous exposure to gemcitabine for 6 days, as previously described [25]. The pancreatic cancer cell lines Capan-1 and MiaPaCa-2 were purchased from the American Type Culture Collection (ATCC^®^, HTB-79™ and ATCC^®^ 1420, Manassas, VA, USA), while PDAC-3 is a primary human PDAC cell culture obtained from resected patients, as described previously [26]. Cells were maintained in RPMI medium (Lonza, BioWhittaker^®^, Basel, Switzerland) supplemented with 10% heat-inactivated foetal calf serum (Biowest, Nuaillé, France) and 1% penicillin/streptomycin (Lonza) and grown at 37 °C, at 5% CO_2_ in a humidified incubator. PANC-1R were cultured in drug-free medium, tested for resistance prior to experiments, and when needed, a pulse treatment of gemcitabine was provided to select the resistant cells. Cells were tested monthly for mycoplasma contamination using the MycoAlert Mycoplasma Detection kit (Westburg, Leusden, The Netherlands), while the identity of the cells was tested yearly by PCR profiling using short tandem repeats (STR).

Substrates of controlled different stiffnesses for two-dimensional (2D) growth were obtained by using hydrogel-coated (Softwell^®^, Cambridge, UK) plates, with a stiffness of 0.5 and 50 kPa.

### 2.2. Evaluation of ITGA2 mRNA Expression in Human Normal and Tumor Tissues and Correlation with Survival in PDAC

The mRNA expression of ITGA2 was evaluated using the web-based genomics analysis and visualization platform GEPIA, analysing the RNA sequencing expression data of 9736 tumors and 8587 normal samples from the TCGA and the GTEx projects (Supplementary Figure S1). Moreover, we performed a correlation of ITGA2 mRNA expression with overall survival using the TCGA-PAAD data of pancreatic cancer specimens.

### 2.3. Evaluation of ITGA2 mRNA and Protein Expression in Two Independent Cohorts of PDAC Ppatients

The primary PDAC tumors of two independent cohorts of PDAC patients were obtained by pancreatico-duodenectomy, total- or distal-pancreatectomy procedures. At the moment of surgery, all patients were treatment-naïve. Following surgery, gemcitabine-monotherapy adjuvant treatment was provided as follows: gemcitabine 1000 mg/m^2^/day on days 1, 8 and 15, every 28 days. The expression of ITGA2 was firstly evaluated by reverse transcription quantitative real-time PCR (RT-qPCR) in a cohort of 85 patients from Mashhad University Hospital, within a study approved by the local Hospital Ethic Committees of the Mashhad University of Medical Sciences with protocol number IR.MUMS.MEDICAL.REC.1400.709. Because of the long experience in previous studies with laser-microdissection methodologies, there was no difficulty in selecting areas with morphological defined cancer cells [27]. Total RNA was successfully extracted using a High Pure RNA Isolation kit (Roche, Mannheim, Germany) and its yields and purity were evaluated at 260–280 nm with a NanoDrop^®^-1000-Detector (NanoDrop-Technologies, Wilmington, NC, USA). RNA (500 ng) was reverse-transcribed using the cDNA synthesis kit Takara Bio (San Jose, CA, USA), according to the manufacturer’s instructions. The resulting cDNA was amplified using specific primers: ITGA2-Forward: GGGAATCAGTATTACACAACGGG; ITGA2-Reverse: CCACAACATCTATGAGGGAAGGG (Macrogene Co., Seoul, Republic of Korea) and SYBR green master mix (Parstous Co., Tehran, Iran). The real-time quantitative PCR reactions were performed in the ABI-PRISM StepOne instrument (Applied Biosystems, ThermoFisher Scientific, Waltham, MA, USA) and data were normalized with the ΔCt calculation using the housekeeping gene GAPDH, whose expression values were the closest to the geometric mean values obtained in preliminary analysis with three housekeeping genes (β-actin, GAPDH and β2-microglobulin).

After checking in 10 PDAC samples that the mRNA expression levels correlated to the protein expression levels, we evaluated the protein expression of ITGA2 by immunohistochemistry in a cohort of 96 patients undergoing surgery at the University Hospital of Pisa. All specimens were obtained after the patient’s written consent was approved by the Ethics Committee of Area Vasta Nord Ovest (CEAVNO, protocol code 724). In this cohort, tissue-microrray (TMA) sections were constructed using core tissue biopsies (diameter 1 mm) obtained from individual paraffin-embedded specimens using the TMA Grand Master instrument (3DHistech, Budapest, Hungary). Before staining, such TMA slides were deparaffinized using xylene, and rehydrated in alcohol. Immunostaining was performed by the avidin–biotin peroxydase complex technique (EnVision System, Dako). Negative controls were obtained by replacement of primary antibody with buffer (PBS 1X). Staining with the recombinant monoclonal ITGA2 antibody (#133557, Abcam, Cambridge, UK; dilution 1:100) was evaluated using a system including both positive cells’ number and intensity. In particular, protein expression was scored by two blind, independent observers, who also evaluated the amount of tissue loss, background staining and overall interpretability. Immunostaining intensity was classified as described in Supplementary Figure S2. According to the observed distribution, the median was chosen as a cut-off and samples were defined as “high ITGA2” when the staining score was > the median, and “low ITGA2” when the staining score was ≤ the median. Neoplastic cells were always uniformly stained and a positivity assessment was made by counting all the tumor cells present in each spot. The same criteria were used to evaluate ITGA2 protein expression in additional TMAs which were constructed with tissue biopsies obtained from individual paraffin-embedded specimens of 45 metastatic patients, before gemcitabine monotherapy palliative treatment, enrolled within the above-mentioned study.

### 2.4. Evaluation of PANC-1 and Its Gemcitabine-Resistant Clone (PANC-1R) by RNA-Sequencing and Label-Free Proteomics

RNA-sequencing of PANC-1 and PANC-1R samples was performed with the Illumina TruSeq Stranded mRNA Library Prep LT kit (RS-122-2201, Illumina Inc., San Diego, CA, USA) and Agencount AMPure XP beads (Beckman Coulter, Brea, CA, USA) using Single-end, 100 bp-reads on the Illumina RAPID Chip on HiSeq 2500 System (Illumina). Bioinformatic pipeline for data analysis was established in a previous study [28,29]. Briefly, FASTX Toolkit (version 0.7) was used for the preprocessing of the raw data, for quality-filtering and adapter-trimming. Sequences were mapped on a human genome (GRCh38) using a STAR alignment tool (version 2.5.3a). The CuffLinks algorithm was used to compute the gene counts in FPKM normalization and plots were created using R version 3.5.0, as described previously [30].

Proteomic analysis of PANC-1 and PANC-1R was performed as previously described [31]. Briefly, lysates of the cell lines were prepared using urea buffer containing HEPES and phosphatase inhibitors. After sonication on ice, protein concentration was quantified with the Bicinchoninic Acid method (ThermoPierce, Waltham, MA, USA). After quantification, proteins were digested and extracted with the whole-in-gel protocol. Finally, samples were analysed through Nano-LC-MS/MS, and protein identification was followed by label-free protein quantification, as previously described [31]. Differential expression of proteomic data were analysed with the beta-binomial test and fold changes were expressed per group comparison. The MS proteomics data were deposited to the ProteomeXchange Consortium via the PRIDE19 partner repository with the dataset identifier PXD010112.

### 2.5. RT-qPCR, Western Blot and Immunocytochemistry of Cells Growing as Monolayer or in 3D

RT-qPCR was used to evaluate gene expression of ITGA2, as described above for the PDAC specimens of the first cohort, as well as to evaluate the gene expression of two genes which have been linked to PDAC invasion and metastasis [32], matrix metalloproteinase-2 (MMP2) and chemokine (C-X-C motif) receptor 4 (CXCR4), using the specific TaqMan^®^ gene expression assays (Hs00234422_m1, and Hs00976734_m1). GAPDH or β-actin were used as the housekeeping gene. Total RNA from 3D culture was isolated using the TRIzol reagent (ThermoFisher Scientific, Waltham, MA, USA), following the manufacturer’s instructions. RNA yields, integrity and purity were checked by measuring optical density at 260–280 nm with a Nanodrop^®^ spectrophotometer. One µg of RNA was used for cDNA synthesis and PCR with the First-Strand cDNA synthesis kit (K1612, ThermoFisher Scientific). The PCR reactions were performed in a 7500 real-time PCR system (Applied Biosystems). The program cycle used started with initial denaturation for 5 min at 95 °C, followed by 40 cycles of 15 s at 95 °C and 60 s at 60 °C. A melt curve was provided after the process finished. The data were normalized with the ΔCt calculation using the housekeeping gene GAPDH or β-actin.

Western blot samples were prepared by whole cell lysis, and 40 µg of protein separated on Mini-Protean TGXTM Precast gels (Bio-Rad laboratories, Hercules, CA, USA) as previously described [33]. After transfer on PDVF (GE Healthcare Bio-Sciences, Uppsala, Sweden) membranes were incubated overnight at 4 °C with rabbit ITGA2 (#133557, Abcam; dilution 1:1000) and rabbit β-actin (#4967, Cell Signaling Technology, Danvers, CO, USA; dilution 1:1000) antibodies. Following primary antibody incubation, secondary anti-rabbit HRP-conjugated (#7074, Cell Signaling Technology, Danvers, CO, USA; dilution 1:2000) antibody was incubated for 1 h at room temperature and protein bands were visualized using enhanced chemiluminescence (ECL).

For the immunocytochemistry analyses, cells grown for 48 h on Softwell^®^ plates or flat-bottom plastic plates were washed with phosphate buffer saline (PBS) and fixed with 4% paraformaldehyde (PFA) for 15 min. After a second wash, the above-mentioned primary antibody against ITGA2 was added (dilution 1:100) and incubated overnight at 4 °C. Primary antibody excess was washed away and a secondary biotinylated antibody was added for 1 h at room temperature. Finally, a solution of alkaline–phosphatase-conjugated streptavidine (1:100 in PBS/1% BSA/0.1% Na-azide, for 30 min; Dako, Glostrup, Denmark) was added to each well and visualized, incubating it in New Fuchsin/naphtol ASBI phosphate solution supplemented with levamisole. Mayer’s Haematoxylin Solution (Merck, Darmstadt, Germany) was used to counterstain cells [34].

### 2.6. Drug Sensitivity, Genetic Knockdown and Overexpression Experiments

Drug sensitivity was assessed using the sulforhodamine B (SRB) assay, as described previously [28]. Gemcitabine was kindly gifted by Eli Lilly Corporation (Indianapolis, IN, USA) and dissolved in sterile water. Briefly, PANC-1, Capan-1 and PDAC-3 were seeded in a 6-well Softwell^®^ plate or 6-well flat-bottom plates at a density of 30,000–50,000 cells/well, while MiaPaCa-2 and PANC-1R were seeded in 96-well flat-bottom plates at a density of 3000 cells/well. The inhibitory concentration of 50% of the cells’ proliferation (IC50) was determined by non-linear least squares curve-fitting with Graphpad Prism (version 7.0, Intuitive Software for Science, Sunnyvale, CA, USA).

Genetic knockdown experiments were performed with ITGA2 Dharmacon siRNA Smartpool comprised of four different siRNAs (Dharmacon, Landsmeer, The Netherlands), and GAPDH Dharmacon siRNA Smartpool (Dharmacon, Landsmeer, The Netherlands) as a negative control, according to the manufacturer’s instructions. Cells were transfected at the final concentration of 50 nM. After 24 h of transfection the medium was refreshed and the cells were incubated for an additional 48 h for the SRB assays, as described above, or for 24 h for the wound-healing, invasion, apoptosis and in vivo experiments, as detailed in the following paragraphs.

ITGA2 was overexpressed in MiaPaCa-2 cells, which has low ITGA2 mRNA levels, using lentivirus-ITGA2 purchased from Genechem (Genechem, Shanghai, China). Cells were transfected with 1 × 10^7^ lentivirus-transducing units with polybrene according to the manufacturer’s instructions. Empty lentiviral vectors were also transfected as a negative control. The cells were then collected and used for in vitro and in vivo studies.

### 2.7. Wound-Healing and Invasion Assay in 3D Collagen Matrix

Analyses of cell motility were performed with the PANC-1, PANC-1R and PDAC-3 cells, using both a wound-healing assay and an invasion assay in 3D collagen matrix, as described previously [35,36].

The wound-healing assay was performed on cells seeded in 6-well Softwell^®^ plates with stiffness of 0.5 kPa or 50 kPa or on flat-bottom 6-well plates. To create a confluent monolayer, a seeding density of 30,000 cells/well was chosen. A removable coverslip glass (Ibidi, Gräfelfing, Germany) was placed in each well to prevent cell attachment in that field. After overnight incubation to let the cells attach to the substrates, the insert was removed and the wells washed with PBS to remove detached cells. Subsequently, medium was added and invasion of the empty area was monitored by taking brightfield images with Universal Grab 6.3 digital software (Digital Cell Imaging Labs, Keerbergen, Belgium) on a Leica DMI300B microscope (Leica Microsystems, Eindhoven, The Netherlands) at various time points (t = 8, 20, 24 h). Finally, the images were analysed using Scratch Assay 6.2 software (Digital Cell imaging Labs).

For the invasion assay in a 3D collagen matrix, we used rat-tail collagen type I isolated by rat tails by acid extraction, as previously described [37]. Collagen gels were created by mixing collagen type I (5 mg/mL), DMEM (Gibco, ThermoFisher Scientific, Waltham, MA, USA), HEPES 0.1 M (1M stock, Biosolve, Valkenswaard, The Netherlands) and NaHCO3 44 mM (440 mM stock, Merck, Darmstadt, Germany) as previously described [38,39] to reach the final concentrations of 0.5 mg/mL (soft) and 2 mg/mL (stiff). The gels were polymerized in 96-well µclear^®^ plates (Greiner Bio-one, Alphen aan den Rijn, The Netherlands) at 37 °C for 1 h before cell injection. To monitor 3D invasion, tumor spheroids were created by automated injection of cell suspensions into the collagen gels using the injection robotics from Life Science Methods (Leiden, The Netherlands). After injection, spheroids were incubated with the appropriate medium at 37 °C. Images of the tumor spheroids were acquired using a Nikon TE2000 confocal microscope equipped with a Prior stage and temperature and CO_2_-controlled incubator. The microscope was controlled through NIS Element Software (Nikon Instruments Inc., Melville, NY, USA). Images were acquired after 2 and 48 h post-injection, with a 20× objective, using the transmission detector of the microscope.

### 2.8. Apoptosis Assay

PANC-1 and PANC-1R cells were treated with IC50 gemcitabine concentrations and with the ITGA2 targeting siRNA pool or the GAPDH pool as negative control for 48 h. At the end of the incubation, cells were washed twice with PBS and fixed in 4% buffered PFA for 15 min. After resuspension, cells were incubated again for 15 min in a Hoechst-33258/bisbenzimide HCl solution (8 µg/mL). Then, drops of cells were spotted on glass slides and visualized by fluorescence microscopy (Leica, Wetzlar, Germany). Two hundred cells were counted in random fields and the apoptotic index was calculated as the percentage of cells showing chromatin condensation and nuclear fragmentation, relatively to the total number of counted cells, as previously described [40]. Active caspase-3 was evaluated in PANC-1 and PANC-1R cells, either untreated or treated with gemcitabine, by using a Quantikine ELISA kit (R&D Systems, Inc., Minneapolis, MN, USA) (Supplementary Figure S9) as previously reported [41].

### 2.9. Analysis of Phospho-AKT by Enzyme Linked Immunosorbent (ELISA) Assay

To investigate the potential modulation induced by ITGA2 overexpression on AKT [pS473] phosphorylation, we performed specific ELISA assays using a specific AKT Colorimetric ELISA kit (Thermo Scientific, Rockford, IL, USA). The levels of phospho-AKT were measured in MiaPaCa-2 cells seeded in a 96-well plate at a density of 10^5^ cells per well, treated for 24 h with gemcitabine at IC50 values. The absorbance was measured in a microplate reader at a wavelength of 450 nm, as described previously [23].

### 2.10. In Vivo

In vivo experiments were performed in athymic female mice following the regulations approved by the local committees on animal experimentation of Mashhad University of Medical Sciences, Mashhad, Iran (protocol number IR.MUMS.MEDICAL.REC.1401.430). PDAC cells (2.5 × 10^6^ PANC-1/PANC-1R cells or 3 × 10^6^ MiaPaCa-2 cells) were suspended in sterile PBS and injected into the flanks of 6-week-old mice. Three to six days after inoculation, when tumor sizes were at least 100 mm^3^, mice were stratified into groups with comparable mean tumor volumes. The mice were treated with gemcitabine at 100 mg/kg, q3dx4, that is, on days 1 and 4, for 3 weeks as previously reported [26]. The body weight and tumor volume were monitored twice per week.

### 2.11. Statistical Analysis

The mRNA and protein expression data were generated blinded to clinical outcomes and the stratification was adopted at the end of the study, considering that in the two cohorts we had more than 85 PDAC patients, with good variability of expression levels, who can be categorized in two equal gene expression subgroups with respect to the median value (i.e., low and high expression groups). Statistical significance was set at *p* < 0.05.

Clinicopathological data of patients were obtained from electronic patient records and referral hospitals and survival data were also checked from regional registries. Disease-free survival (DFS) was defined as the time from the diagnosis to the first relapse or last follow-up in radically resected patients. Progression-free survival (PFS) was defined as the time from the diagnosis to the first progression or last follow-up in metastatic patients. Overall survival (OS) was calculated from the diagnosis to the death or last follow-up. Kaplan–Meier curves were generated to visualize OS and PFS, which were then evaluated by log-rank test. Univariate analysis was performed and factors with a *p*-value below 0.1 were evaluated in the multivariate analysis using Cox’s proportional hazards model. Data were analysed using SPSS^®^ Statistics version 23 statistical software (IBM, Chicago, IL, USA). Statistical significance was set at *p* < 0.05.

In vitro studies were performed in triplicate and repeated in at least two independent experiments. Data are expressed as means ± SEM if not specified otherwise. If not specified otherwise, Graphpad Prism version 7 was used to create plots. A two-sided t-test was used to analyse statistical significance (with significance * *p* < 0.05, ** *p* < 0.01, *** *p* < 0.001, **** *p* < 0.0001).

## 3. Results

### 3.1. High Expression Levels of ITGA2 Are an Unfavorable Prognostic Factor in Gemcitabine-Treated Patients

The role of integrin α2 (ITGA2) expression as a potential prognostic factor was first checked on publicly available databases (i.e., GEPIA). ITGA2 is highly expressed in pancreatic cancer tissues and correlates with a decreased overall survival (OS) and disease-free survival (DFS) rate in patients with high ITGA2 expression (Supplementary Figure S1).

To investigate whether the ITGA2 prognostic value was confirmed in cohorts of patients treated with gemcitabine-based chemotherapy, we evaluated ITGA2 expression by RT-qPCR and immuno-histochemistry in two independent cohorts of radically resected PDAC patients (n = 86 in the first cohort and n = 96 in the second cohort) who received gemcitabine either as adjuvant therapy, as well as a small cohort of metastatic patients (n = 45) who received gemcitabine as palliative therapy. No significant differences in OS and DFS were observed between the first and second cohorts, underlying the homogeneity of these two cohorts and of the gemcitabine-based adjuvant treatments. The clinicopathological characteristics and their accordance with OS and DFS in these cohorts are described in Table 1 and Table 2 and Supplementary Figure S3.

No significant difference in OS and DFS was observed considering the clinicopathological features of the patients, except ITGA2 mRNA expression. ITGA2 levels showed variability among patients and no significant correlations with demographic/clinical characteristics. No discrepancies were observed in the samples analysed in duplicate (approximately 10%), and strong staining for ITGA2 was associated with shorter OS. Indeed, patients with ITGA2 expression above the median had a significantly shorter OS (median OS 13.6 vs. 20.9 months, log-rank test *p* = 0.042). Similar results were observed for DFS, with high ITGA2 expression correlating with significantly shorter DFS in this cohort (log-rank test *p* = 0.045).

The protein expression pattern of ITGA2 in the second cohort of patients was also correlated to the clinical outcome. High expression of ITGA2 was correlated to decreased OS (median OS 16.1 vs. 20.6 months, log-rank test *p* = 0.014) and DFS (log-rank test *p* = 0.010). High or low expression of ITGA2 was not correlated to other clinicopathological features. However, among the other clinicopathological characteristics, the histological grading showed a trend toward a significant correlation to OS and DFS, with shorter OS and DFS in patients with grade 3 compared to patients with grade 1 or 2 (log-rank test *p* = 0.06 and *p* = 0.10). At the multivariate analysis, ITGA2 emerged as an independent prognostic factor for OS. In particular, high expression levels of ITGA2 were associated with an increased risk of death (HR = 1.7, 95%CI, 1.1–2.6, *p* = 0.01), and a trend toward a statistically significant increase of relapse (HR = 1.5, 95%CI, 0.9–2.1, *p* = 0.07).

**Figure 1:**
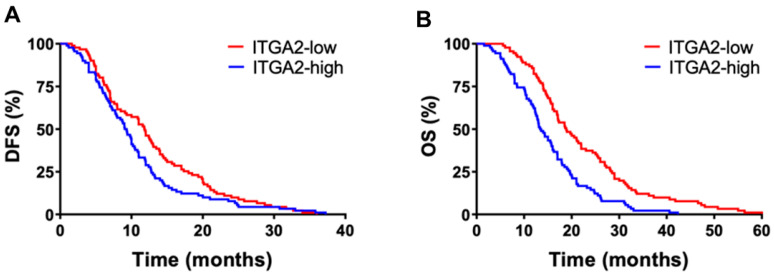
Prognostic value of ITGA2. Kaplan–Meier curves of the patients in the first and second cohort (n = 181) grouped according to ITGA2 expression, showing a reduced DFS (**A**) and OS (**B**) in patients with high (blue) vs. low (red) expression of ITGA2.

Then, we performed a statistical analysis on all the 181 subjects, and Figure 1 shows the OS and DFS curves pooling the patients from the first and second cohort grouped according to ITGA2 high/low expression vs. median values. Patients with high ITGA2 protein expression had a significantly shorter mean DFS (8.5 vs. 11.1 months, *p* = 0.03) compared to patients with low ITGA2 expression (Figure 1A). A stronger statistical difference was observed when evaluating OS, with mean values of 14.9 months vs. 20.7 months, in patients with high vs. low ITGA2 expression (*p* < 0.01), respectively (Figure 1B). Additionally, since all patients were treatment-naïve, we concluded that the increased ITGA2 expression observed was associated with an inherent primary resistance.

Finally, we evaluated the expression of ITGA2 in a small cohort of metastatic patients (n = 45) and showed that the high ITGA2 expression group had a poorer prognosis than the low expression group (Supplementary Figure S3). Patients with ITGA2 expression above the median had a significantly shorter mean OS (7.6 months; 95% confidence interval (95% CI), 5.3–10.1 months) compared with patients with ITGA2 expression lower than the median (13.8 months; 95% CI, 10.6–16.9 months; *p* = 0.01). Similar significant results (*p* = 0.03) were obtained with the PFS curves of patients with ITGA2 expression above the median, with a median of 5.7, compared with 8.6 months in patients with low ITGA2 expression.

### 3.2. High Expression Levels of ITGA2 Are Associated with Gemcitabine Resistance and Stiffness

**Figure 2:**
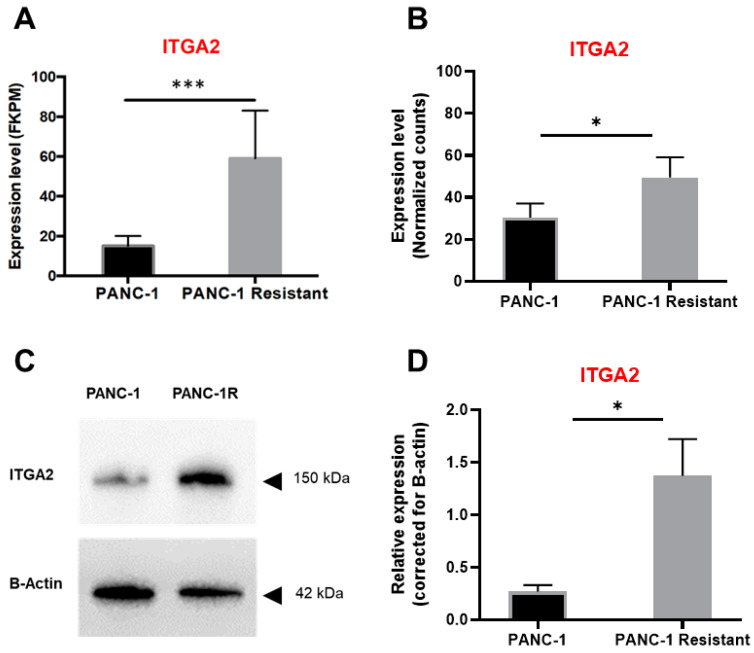
Gemcitabine-resistant cells have increased ITGA2 expression. (**A**) ITGA2 expression, reported as fragments per kilo base of transcript per million mapped fragments (FKPM), from RNA-sequencing of PANC-1 and PANC-1R cell lines (in black and grey, respectively). Columns, mean values; bars, SD. *** *p* < 0.001. (**B**) ITGA2 expression, reported as a fold-change of normalized counts, from label-free proteomics of PANC-1 and PANC-1R cell lines (in black and grey, respectively). Data are reported as mean ± SD of two experiments performed in duplicate. * *p* < 0.05. (**C**) Western blot of ITGA2 expression in PANC-1 and PANC-1R and (**D**) relative quantification normalized to loading control. * *p* < 0.05. Original, uncropped Western blot membrane can be found in Supplementary Figure S5.

To investigate the link between ITGA2 overexpression and gemcitabine resistance, we employed a stable gemcitabine-resistant cell line (PANC-1R), obtained from the wild-type cell PANC-1, as described previously [25]. These cells were analysed by RNA-sequencing and label-free proteomic. Remarkably, both omics analyses reported that ITGA2 was overexpressed in PANC-1R cells (Figure 2A,B). ITGA2 overexpression in PANC-1R cells was further confirmed at protein level via Western blot analysis (Figure 2C,D) and FACS (Supplementary Figure S4). Other “classical determinants” of gemcitabine activity (i.e., the human equilibrative nucleoside transporter-1, the activating enzyme deoxycytidine kinase and the target ribonucleotide reductase [42]) were not significantly modulated in the PANC-1R cells, as previously reported [31].

**Figure 3:**
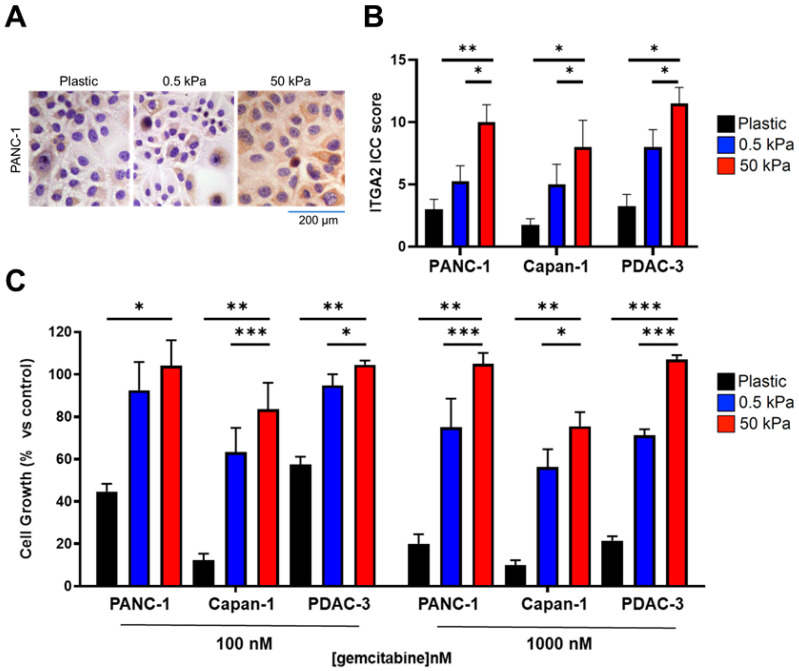
Stiffness increases ITGA2 expression and triggers gemcitabine resistance. (**A**) ITGA2 expression level analysed by immunocytochemistry (ICC). Representative image of ICC staining of PANC-1 cells growing for 48 h either on uncoated plastic or on soft and stiff collagen-coated bis-acrylamide gel (0.5 kPa and 50 kPa). (**B**) ITGA2 ICC score for PANC-1, Capan-1 and primary PDAC-3 cells growing on uncoated plastic (black), soft gel (0.5 kPa, blue) or stiff gel (50 kPa, red). Data are reported as mean ± SD of two experiments performed in duplicate. * *p* < 0.05, ** *p* < 0.01 (**C**) Cell survival percentage comparing gemcitabine treatment to control untreated cells as assessed by SRB assay. Survival % of PANC-1, Capan-1 and primary PDAC-3 cell lines growing on uncoated plastic (black), soft gel (0.5 kPa, blue) or stiff gel (50 kPa, red) after exposure to gemcitabine for 72 h. Data are reported as mean ± SEM of four experiments performed in duplicate. * *p* < 0.05, ** *p* < 0.01, *** *p* < 0.001.

Given the involvement of integrin α2β1 in mediating cellular interactions with collagen-rich ECM, we decided to study the behavior of cells growing on collagen-coated bis-acrylamide gel plates of tunable stiffness (i.e., 0.5 and 50 kPa). These stiffness values were selected to mimic the pancreatic tissue physiological and pathological stiffnesses, with 0.5 kPa representing healthy pancreatic tissue, and 50 kPa for advanced PDAC [9]. As a control, cells were grown on uncoated plastic. By immunocytochemistry, the expression of ITGA2 was evaluated in response to changes in substrate stiffness (Figure 3A,B). When growing on soft (0.5 kPa) and even more on stiff (50 kPa) substrates, all the PDAC cells significantly increased ITGA2 expression (Figure 3A,B and Supplementary Figure S6), highlighting the importance of mechanosensing. Most interestingly, stiffness affected cells’ sensitivity to gemcitabine. Surprisingly, all cells exposed to cytotoxic concentrations of gemcitabine (i.e., 100 and 1000 nM) had a decreased survival rate when growing on plastic, but became resistant to gemcitabine when seeded on collagen-coated soft and stiff gels (Figure 3C). More precisely, PANC-1, Capan-1 and PDAC-3 growing on plastic had a survival rate of 40% and 20% when exposed to 100 and 1000 nM gemcitabine, respectively. However, when on soft, and even more on stiff gels, all cells showed an increased survival rate ranging from 80% to 100%. These results indicate that the presence of collagen, which triggers ITGA2 activation, along with substrate stiffness play a crucial role in gemcitabine resistance.

### 3.3. Substrate Stiffness Influences Invasive Behavior in 2D and 3D

**Figure 4:**
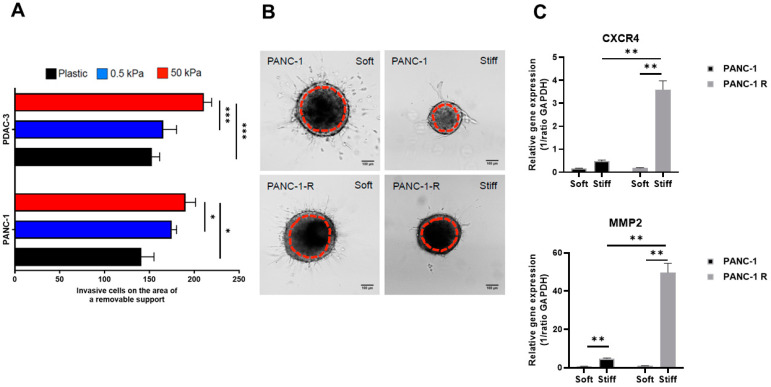
Substrate stiffness in 2D and 3D invasion. (**A**) The 2D cell migration of PANC-1 and primary PDAC-3 cell lines growing on uncoated plastic (black), soft gel (0.5 kPa, blue) or stiff gel (50 kPa, red) as assessed by wound healing assay after 24 h. Data are expressed as mean ± SD of two experiments performed in duplicate. * *p* < 0.05, *** *p* < 0.001. (**B**) Representative images of PANC-1 and PANC-1R spheroids embedded in soft (0.5 mg/mL) or stiff collagen (2 mg/mL). Brightfield images show spheroids’ size 2 days post-injection, while the dashed red line represents the initial spheroids’ size 2 h post-injection. Scale bar = 100 μm. (**C**) Gene expression level of CXCR4 and MMP2 in 3D spheroids, as assessed by RT-qPCR. RNA was extracted from PANC-1 (black) and PANC-1R (grey) spheroids embedded in soft (0.5 mg/mL) or stiff collagen (2 mg/mL). Columns, mean values; bars, SD. ** *p* < 0.01.

Since substrate stiffness and ECM composition influence cellular motility and invasion, which have been related to chemoresistance [43], we investigated cell migration and invasion in 2D and 3D models. By a wound healing assay, we observed that in the 2D setting, both immortalized and primary cell lines (PANC-1 and PDAC-3, respectively) migrated more on stiff than on soft gels and on uncoated plastic (Figure 4A). Representative immunofluorescence pictures obtained by intracellular staining with propidium iodide are reported in the Supplementary Figure S7.

However, when cells were grown as 3D collagen-embedded spheroids, their behavior was different. In contrast to 2D migration, PANC-1 cells were less invasive in stiff gels, and spheroid growth was constrained, as can be seen in the brightfield images 2 days after injection (Figure 4B). The same behavior was observed for PANC-1R cells. Two interesting matrix remodeling genes are MMP2 and CXCR4, which are involved in promoting cell motility and invasion in pancreatic cancer [44,45]. Additionally, collagen I and ITGA2 expression can influence MMP2 expression and activity [46,47]. Therefore, to assess whether PANC-1R would respond differently from PANC-1 to the microenvironment, we extracted RNA from spheroids 2 days post-injection and evaluated the mRNA expression of MMP2 and CXCR4. Remarkably, only PANC-1R cells growing in stiff gels showed a significant increased expression of matrix remodeling genes (Figure 4C), indicating that a stiffer collagen matrix triggers a more invasive phenotype of gemcitabine-resistant cells.

### 3.4. ITGA2 Expression Levels Modulate Gemcitabine Sensitivity In Vitro

**Figure 5:**
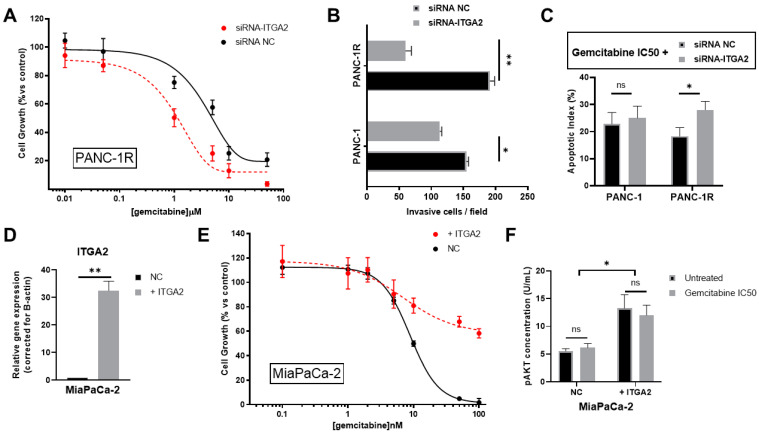
Role of ITGA2 in gemcitabine sensitivity in vitro. (**A**) Growth curves of PANC-1R cells exposed for 48 h to gemcitabine with concentrations ranging from 0.1 to 10 μM and transfected with siRNA-GAPDH (black = negative control, siRNA NC) or siRNA-ITGA2 (red), as assessed by SRB assay. Data are expressed as mean ± SEM of three experiments performed in duplicate. (**B**) Migration of PANC-1 and PANC-1R cells transfected with siRNA-GAPDH (black = negative control, siRNA NC) or siRNA-ITGA2 (grey), as assessed by wound healing assay after 24 h. Data are expressed as mean ± SEM of three experiments performed in duplicate. * *p* < 0.05, ** *p* < 0.01. (**C**) Apoptotic index of PANC-1 and PANC-1R cells treated with gemcitabine at IC50 values, and transfected with siRNA-GAPDH (black = negative control, siRNA NC) or siRNA-ITGA2 (grey). Data are expresses as mean ± SD of three experiments performed in duplicate. ns = not significant, * *p* < 0.05. (**D**) ITGA2 mRNA expression in MiaPaCa-2 cells transfected with empty vector (black = negative control, NC) or ITGA2-overexpressing vector (grey = +ITGA2), as assessed by qRT-PCR. Data are expressed as mean ± SD of two experiments performed in duplicate. ** *p* < 0.01. (**E**) Growth curves of MiaPaCa-2 cells exposed for 72 h to gemcitabine with concentrations ranging from 0.1 to 100 nM and transfected with empty vector (black = negative control, NC) or ITGA2-overexpressing vector (red = +ITGA2), as assessed by SRB assay. Data are expressed as mean ± SD of two experiments performed in triplicate. (**F**) Phospho-AKT levels (U/mL) measured by ELISA in MiaPaCa-2 negative control (NC) or ITGA2-overexpressing (+ITGA2) cells either untreated (black) or treated with gemcitabine IC50 (grey). ns = not significant, * *p* < 0.05.

In order to evaluate whether ITGA2 overexpression in chemoresistant cells has a pivotal role on the aggressive phenotype, we silenced this gene via siRNA, as assessed by RT-qPCR and Western blot (Supplementary Figure S8). Interestingly, after ITGA2 knockdown, chemoresistant cells PANC-1R were more sensitive to gemcitabine, as assessed via the SRB assay which reported an IC50 of 1.0 µM for siRNA-ITGA2 and an IC50 of 5.2 μM for the non-targeting control (Figure 5A). We further analysed the migratory potential in the 2D wound healing assay, where we observed a statistically significant reduction of migration in siRNA-ITGA2-treated PANC-1R cells (which overexpress ITGA2), and only a small effect on silenced PANC-1 parental cells (Figure 5B). Additionally, apoptosis caused by exposure to gemcitabine was increased in siRNA-ITGA2 PANC-1R cells (Figure 5C and Supplementary Figure S9).

To validate our findings that ITGA2 overexpression is responsible for gemcitabine resistance in PANC-1R cells, we overexpressed ITGA2 in a gemcitabine-sensitive cell line (MiaPaCa-2) that expresses very low mRNA levels of ITGA2 and does not express the ITGA2 protein, as reported previously [48]. ITGA2 overexpression was assessed by RT-qPCR, which showed a 32-fold increase compared to the negative control (Figure 5D). In these MiaPaCa-2 cells overexpressing ITGA2 we observed a significant reduction of sensitivity to gemcitabine, with a shift of IC50 from 9 nM to > 100 nM in MiaPaCa-2 negative control and ITGA2-overexpressing cells, respectively (Figure 5E).

Previous studies showed that ITGA2 overexpression increased cell proliferation and reduced apoptosis by upregulating phospho-AKT (pAKT) in ovarian and esophageal cancer [17,18]. In addition, increased pAKT levels have been associated with gemcitabine resistance in PDAC cells and patients [23]. We therefore measured the levels of pAKT in our ITGA2-overexpressing MiaPaCa-2 cells, and found a significant increase compared to the negative control, regardless of treatment with gemcitabine (Figure 5F). Altogether, these results support our hypothesis that ITGA2 plays a pivotal role in mediating gemcitabine sensitivity/resistance in PDAC cells.

### 3.5. ITGA2 Expression Levels Modulate Gemcitabine Sensitivity In Vivo

**Figure 6:**
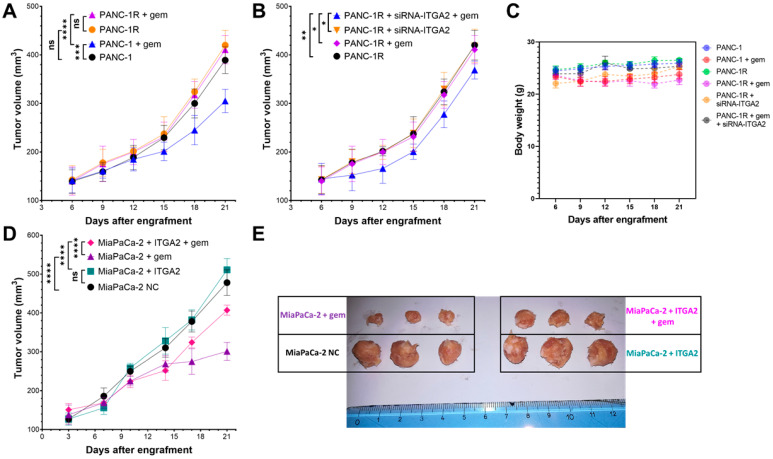
Role of ITGA2 in gemcitabine sensitivity in vivo. (**A**) Tumor volumes of mice subcutaneously injected with PANC-1 (black), PANC-1R (orange), PANC-1 followed by gemcitabine (gem) treatment (blue) or PANC-1R followed by gemcitabine (gem) treatment (purple). Data are expressed as mean ± SD from six tumors. ns = not significant, *** *p* < 0.001, **** *p* < 0.0001. (**B**) Tumor volumes of mice subcutaneously injected with PANC-1R cells (black), PANC-1R transfected with siRNA-ITGA2 (orange), PANC-1R treated with gemcitabine (gem, purple) or PANC-1R + siRNA-ITGA2 in combination with gemcitabine treatment (blue). Data are expressed as mean ± SD from six tumors. * *p* < 0.05, ** *p* < 0.01. (**C**) Body weight of mice monitored for all the aforementioned conditions after injection of PANC-1 or PANC-1R cells. (**D**) Tumor volumes and (**E**) representative tumors’ images from mice subcutaneously injected with MiaPaCa-2 cells transfected with empty vector (black = MiaPaCa-2 negative control, NC), MiaPaCa-2 with ITGA2-overexpressing vector (green = MiaPaCa-2 + ITGA2), MiaPaCa-2 transfected with empty vectors and treated with gemcitabine (purple = MiaPaCa-2 + gem) or transfected with an ITGA2-overexpressing vector followed by gemcitabine treatment (magenta = MiaPaCa-2 + ITGA2 + gem). Data are expressed as mean ± SD from six tumors. **** *p* < 0.0001. In all conditions, gemcitabine was administered intraperitoneally, 100 mg/kg, 2 days a week.

To validate our in vitro results in in vivo models, parental PANC-1 and gemcitabine-resistant PANC-1R were injected into the flank of nude mice, treated with gemcitabine and tumor growth evaluated. As expected, a significant tumor growth inhibition (*p* = 0.0002) was obtained only in PANC-1 tumors treated with gemcitabine (Figure 6A). Contrarily, PANC-1R tumors were inherently resistant to gemcitabine and, as previously reported [31], had a trend towards forming larger tumor compared to PANC-1 (although not statistically significant, *p* = 0.09) (Figure 6A). To investigate whether ITGA2 knockdown could restore chemosensitivity in vivo, ITGA2-silenced PANC1-R were injected into the mice, and subsequently treated with gemcitabine. Remarkably, a significant reduction in tumor growth was observed in this group of animals compared to all the other groups (Figure 6B). In all the conditions evaluated mice body weights were not significantly different (range 22–29 g), suggesting that the treatments were not toxic (Figure 6C).

Finally, to further demonstrate the role of ITGA2 overexpression in gemcitabine resistance in vivo, MiaPaCa-2 cells overexpressing ITGA2 (as assessed by RT-qPCR, Supplementary Figure S10) or MiaPaCa-2-negative control cells, were subcutaneously injected, and mice treated with gemcitabine. In keeping with the above-mentioned results, we did not observe a significant difference in tumor growth between negative control and ITGA2-overexpressing tumors (Figure 6D,E). The treatment with gemcitabine reduced the tumor volumes. However, MiaPaCa-2 ITGA2-overexpressing tumors treated with gemcitabine were significantly more resistant compared to MiaPaCa-2 negative control tumors treated with gemcitabine (*p* < 0.0001, Figure 6D,E).

## 4. Discussion

In this study, we evaluated the role of integrin α2 (ITGA2) as a novel prognostic factor for PDAC, as well as its association to resistance to gemcitabine and mechanical cues. Patients with radically resectable and metastatic unresectable PDAC who had high expression of ITGA2 showed a significantly shorter survival. All these patients were treated with gemcitabine, in the adjuvant or palliative setting, prompting further studies to unravel the role of ITGA2 in therapy resistance. Therefore, we evaluated the gemcitabine-resistant cells (PANC-1R), which showed increased expression of ITGA2 at both the RNA and protein levels. Additionally, we observed how overexpression of ITGA2 was induced by matrix stiffness, and associated to gemcitabine resistance for both 2D and 3D model systems that simulate the natural stiffness range found in PDAC. Finally, we reported that overexpression of ITGA2 in gemcitabine-sensitive cells (MiaPaCa-2) induces chemoresistance and cancer cell growth both in vitro and in vivo. The aggressive chemoresistant phenotype was reverted by silencing of ITGA2.

Integrin α2β1 is a protein involved in ECM-cellular mechanosensing, mainly through collagen type-I binding, which activates a signaling cascade leading to cytoskeleton rearrangements and cellular motility [49,50]. ITGA2 involvement in “stiff” cancers, such as breast, prostate, colon and pancreatic cancer, has been extensively studied [20,22]. In particular, ITGA2 was reported to be a putative biomarker and poor prognostic factor for PDAC patients [21,51,52]. However, to our knowledge, this is the first study investigating its role in resistance to gemcitabine in PDAC. Previous studies analysed ITGA2 expression on publicly available datasets reporting that high ITGA2, along with its hub genes (LAMB3, LAMC2 and FN1), is an unfavorable prognostic factor for PDAC patients [21,22]. Nevertheless, it was not investigated whether patients received a chemotherapy regimen and if there was a correlation between therapy response and ITGA2 expression level. Here, we observed for the first time that not only in radically resected patients but also metastatic patients, who received gemcitabine as palliative care, high ITGA2 expression was correlated to poorer survival rates, indicating that ITGA2 might be responsible for therapy resistance. Our observation should prompt future biomarker-driven perspective trials, aiming at optimal patients’ stratification in preventing therapy resistance. Indeed, patients with high expression of ITGA2 should be treated with regimens that do not include gemcitabine. However, since the present study did not investigate other therapy regimens, we can only speculate that FOLFIRINOX might be an alternative, but that needs to be further validated in the pre-clinical and clinical setting [5].

As reviewed by Kalli and Stylianopoulos, tissue stiffness affects cancer cell growth, tumor progression and cell migration in different tumor types. More specifically, ECM stiffening is sensed by cancer cells through integrins and mechanosensor molecules, which in turn induce cytoskeleton contractility and increased migration [53]. Breast cancer and PDAC are two of the stiffer cancers (up to 50 kPa) and subsequent tissue morphological changes were studied by using both 2D and 3D model systems [13]. However, the involvement of tissue stiffness in chemoresistance is still poorly understood and seems to have controversial and tumor-dependent effects [13,54]. In breast cancer it was reported that a soft environment, rather than a stiff, is responsible for chemotherapy resistance [54]. On the other hand, Rice and collaborators showed that a stiff environment is responsible for increased tumorigenesis and chemoresistance of PDAC [13]. Our results partially agree with findings from Rice and collaborators, showing that chemoresistance increases with matrix stiffness. In contrast to Rice et al., here we reported a clear effect of stiffness in the induction of gemcitabine resistance in PDAC cells. The difference we observed might be explained by the different cell lines used and/or by the lack of an uncoated plastic control. Interestingly, we observed that the cell line BxPC3 (used by Rice et al.) lacks KRAS mutation, found in 94% of all PDAC cancers and responsible for cancer cell growth and proliferation [55], while PANC-1, Capan-1 and PDAC-3 all harbor KRAS mutations [56]. Therefore, we speculate this could alter gemcitabine cytotoxicity and it would be interesting to further validate this by introducing mutated KRAS in wild-type PDAC cells. A noteworthy aspect is that the presence of a collagen coating also triggered the resistance of PDAC cells. Compared to plastic, cells growing on collagen-coated gels showed reduced sensitivity to the drug. This finding suggests that it is not only stiffness, but also the exposure to collagen-induced expression of ITGA2 which made cells resistant to gemcitabine.

A seminal work, investigating the correlation between ECM stiffness and glioma cell migration, reported that on stiffer gels, glioma cells had increased motility and invasion potential [57]. This was further studied in other cancer types, such as breast cancer and PDAC, showing that cancer cells growing on a stiffer substrate have a more stretched phenotype, and increased migration and invasion [12,58,59]. Our results for 2D invasion of PDAC cell lines are in line with previous studies, showing that cell migration increases with stiffness. However, in 3D invasion models, the situation appears to be slightly different. Here, not only the stiffness, but also the matrix composition, density and cross-linking appeared to play an important role. Indeed, solid stress (which comprises the abovementioned features) is known to influence how cells behave in a physiological context. More precisely, increasing the stiffness of 3D matrices can be achieved by either increasing the components’ concentration or the orientation and crosslinking efficiency [53,60]. Here we increased the collagen concentration (0.5 mg/mL and 2 mg/mL) to obtain a stiffer matrix, which inherently increases the solid stress the matrix applies on cancer cells, leading to a physical constraint for invasion. This might partially explain the reason why collagen-embedded 3D spheroids tend to invade more in a soft matrix rather than in stiff ones. Although stiffness and solid stress cannot be separated, it is interesting to note the different behavior of chemoresistant cells. The stably resistant PDAC cell line (PANC-1R) surprisingly exhibited a higher invasive potential, compared to the parental PANC-1. When spheroids were embedded in stiffer collagen, PANC-1R cells strongly increased the expression of the matrix remodeling genes MMP2 and CXCR4. Consistently with the existing literature, stiffer and denser collagen induced an increase of MMP2 expression in both cell lines [61]. Surprisingly, the increased expression of CXCR4 and MMP2 in PANC-1R cells was markedly greater compared to PANC-1 cells, suggesting a different way of adapting to the environmental changes that should be further investigated.

To further elucidate the involvement of ITGA2 in gemcitabine resistance, we performed knockdown via siRNA and investigated drug sensitivity, migration and apoptosis. The reduced migration in siRNA-ITGA2 PANC-1R cells overexpressing ITGA2 is in line with previous studies, which reported a decreased migration in PDAC, gastric and breast cancer cell lines upon silencing of this gene [19]. Remarkably, silencing of ITGA2 was also able to resensitize PANC-1R cells to gemcitabine both in vitro and in vivo [19]. We did not observe a significant modulation of cell proliferation, neither of tumor growth after silencing of ITGA2 in our PANC-1R model. However, we report that significant tumor growth inhibition was achieved by combining ITGA2 knockdown with gemcitabine treatment. Furthermore, we demonstrated that overexpression of ITGA2 in the gemcitabine-sensitive cell line MiaPaCa-2 [62,63] induces gemcitabine chemoresistance both in vitro and in vivo. Regarding the potential molecular mechanism underlying this phenomenon, a previous study reported that ITGA2 silencing inhibits FAK/AKT signaling, thereby decreasing cell proliferation in esophageal squamous cell carcinoma [17]. Moreover, ITGA2 silencing induces apoptosis in gastric cancer [64] and is able to restore sensitivity to paclitaxel in ovarian cancer via inhibition of AKT/FOXO1 signaling [18]. In agreement with these studies, we found that siRNA-ITGA2 triggers apoptosis in PANC1-R cells treated with gemcitabine. Furthermore, ITGA2 overexpression in MiaPaCa-2 cells was associated with a significant increase in AKT activity. Altogether, this evidence supports the fact that a blockade of overexpressing ITGA2 stops aberrant cell proliferation, therefore increasing cytotoxic drugs’ efficacy, as we observed in the present study.

We acknowledge the limitations of this study, as further knowledge on the molecular mechanism of ITGA2-mediated chemoresistance to gemcitabine in PDAC is required, as well as the generation of other PDAC chemoresistant cell lines. Since the increased stiffness of the tumor is mainly mediated by CAFs [65,66], further studies with co-cultures of PDAC-resistant cells and CAFs will deepen our understanding of the role of ITGA2-increased expression in the TME. However, the presented study evaluated a large series of PDAC patients treated with gemcitabine, and shed light on a previously unknown role of ITGA2 (i.e., chemoresistance in PDAC), which is worth further investigation. Of note, ITGA2 is an appealing therapeutic target not only in PDAC, but also in other cancer types [17,18,64]. More precisely, inhibition of ITGA2 would alleviate tumor solid stress, block cancer cell aberrant proliferation, and partially prevent chemoresistance. ITGA2 inhibitors such as bufalin, a natural compound found in toad venom, and E7820, a sulphonamide derivative, already entered the clinical experimentation phase. While both succeeded in phase I, bufalin did not show any beneficial effect in PDAC patients receiving the combination of bufalin + gemcitabine (NCT00837239) [67]. On the other hand, E7820 was investigated in a phase II trial, mainly for colorectal cancer (NCT01133990, NCT01347645), but results are not yet available. Finally, another possible therapeutic approach for ITGA2 would be nanotherapeutics; Guo and collaborators showed that designing ITGA2 antibody-coated nanoparticles containing cytotoxic drugs highly improved drug delivery and cell death in preclinical models of glioblastoma [68].

## 5. Conclusions

In this study, we investigated the prognostic value of ITGA2 and the role of mechanical cues in resistance to gemcitabine in PDAC. Metastatic patients with high ITGA2 expression had a poorer prognosis and resulted to be resistant to gemcitabine treatment. By investigating ITGA2 in in vitro experiments, we showed that it is overexpressed in resistant cell lines and that a stiff environment is also able to induce a substantial increase in ITGA2. The stiffness and increased expression of ITGA2 in turn triggered a significant resistance to gemcitabine. Finally, we demonstrated that modulation of ITGA2 expression, by silencing or overexpression, is responsible for gemcitabine sensitivity both in vitro and in vivo. These results collectively highlight the role that ITGA2 and a stiff environment have in inducing chemoresistance. Increased apoptosis in siRNA-ITGA2 cells treated with gemcitabine and upregulation of phospho-AKT in ITGA2-overexpressing cells suggest a possible molecular mechanism, warranting further evaluation in future studies. However, the present study sheds light on a new role of ITGA2 in PDAC, paving the way for patient therapy stratification based on ITGA2 expression. This could improve chemoresistance and prompts further investigation of ITGA2 as a potential therapeutic target in PDAC.

## Figures and Tables

**Table 1:** Clinicopathological characteristics and correlation with mean overall survival (OS) and disease-free survival (DFS) rates of the PDAC patients of the first cohort.

Univariate Analysis	No, %	OS Months (95% CI)	*p*	DFS Months (95% CI)	*p*
No. Patients	85	21.9 (17.9–25.9)		14.5 (9.3–19.6)	
Age, years					
≤65	40 (47)	17.9 (13.1–22.7)	0.36	9.4 (5.2–13.7)	0.35
>65	44 (53)	23.6 (18.1–29.0)		12.5 (7.7–17.3)	
Sex					
Male	46 (54)	17.7 (13.6–21.8)	0.61	10.7 (6.8–14.5)	0.22
Female	39 (46)	24.5 (18.3–30.7)		11.7 (5.9–17.5)	
Grading					
1–2	32 (38)	22.9 (16.3–29.5)	0.67	12.0 (7.4–16.6)	0.69
3	53 (62)	18.9 (14.9–22.9)		10.3 (5.7–14.9)	
Stage					
IIA	42 (50)	21.4 (15.7–27.1)	0.60	12.0 (7.4–16.6)	0.87
IIB	43 (50)	19.8 (15.4–24.2)		10.3 (5.7–14.9)	
ITGA2 expression					
low	43 (50)	20.9 (16.5–25.1)	**0.042**	10.7 (8.7–18.7)	**0.045**
high	42 (50)	13.6 (6.4–20.7)		8.3 (2.6–14.1)	

Abbreviations: DFS, disease-free survival; OS, overall survival; significant *p*-values are in bold.

**Table 2:** Clinicopathological characteristics and correlation with mean overall survival (OS) and disease-free survival (DFS) rates of the PDAC patients of the second cohort, using both univariate and multivariate analysis.

Univariate Analysis	No. %	OS Months (95% CI)	*p*	DFS Months (95% CI)	*p*
No. Patients	96	18.3 (16.6–20.0)		10.0 (8.8–11.3)	
Age, years					
≤65	45 (47)	18.5 (15.9–21.1)	0.98	10.4 (8.5–12.3)	0.74
>65	51 (53)	18.2 (16.0–20.4)		9.7 (8.0–11.3)	
Sex					
Male	49 (51)	18.4 (16.3–20.5)	0.80	9.9 (7.1–10.7)	0.61
Female	47 (49)	18.2 (15.6–21.0)		11.2 (9.4–12.9)	
Grading					
1–2	52 (55)	19.8 (17.4–22.2)	**0.06**	10.8 (8.9–12.6)	**0.10**
3	43 (45)	16.2 (14.0–18.4)		8.8 (7.3–10.3)	
Stage					
IIA	6 (6)	18.7 (14.5–22.8)	0.81	9.1 (6.0–12.2)	0.57
IIB	90 (94)	18.7 (16.9–20.5)		10.1 (8.8–11.5)	
ITGA2 expression					
low	48 (50)	20.6 (18.1–23.1)	**0.01**	11.4 (9.5–13.4)	**0.04**
high	48 (50)	16.1 (13.9–18.2)		8.7 (7.2–10.1)	
**Multivariate** **analysis**	**Covariates**	**Risk of death**	***p***	**Risk of progression**	***p***
Grading (WHO)	1 vs. 2	1.6 (0.9–2.3)	**0.06**	1.3 (0.8–1.9)	0.19
ITGA2 expression	Low vs. high	1.7 (1.1–2.6)	**0.01**	1.5 (0.9–2.1)	**0.07**

Abbreviations: DFS, disease-free survival; OS, overall survival; reference in the multivariate analysis and significant *p*-values are in bold.

## Data Availability

Raw proteomics data has been deposited on ProteomeXchange under accession number PXD010112 (Le Large et al. 2019) [31]. Raw RNA-sequencing data has been deposited on GEO under accession number GSE207274.

## Supplementary Materials

The following supporting information can be downloaded at: https://www.mdpi.com/article/10.3390/cancers15030628/s1, Figure S1: ITGA2 expression profile and prognostic value in publicly available database; Figure S2: Representative TMA cores and scoring system; Figure S3: Clinicopathological characteristics and Kaplan–Meier curves of the metastatic cohort; Figure S4: Flow cytometry analysis of ITGA2 expression levels on the cell surface of PANC-1 and PANC-1R cells; Figure S5: Uncropped Western blot membrane of PANC-1 and PANC-1R stained for ITGA2 and β-actin; Figure S6: Representative immunocytochemistry images of Capan-1 and PDAC-3 cell lines; Figure S7: Representative immunofluorescence images of the 2D wound healing assay; Figure S8: ITGA2 knockdown efficiency in PANC-1R cells; Figure S9: Apoptosis induction in PANC-1 and PANC-1R cells; Figure S10: ITGA2 overexpression in MiaPaCa-2 cells injected in mice.
