# Supplementary material for: Prognostic Significance of Integrin Subunit Alpha 2 (ITGA2) and Role of Mechanical Cues in Resistance to Gemcitabine in Pancreatic Ductal Adenocarcinoma (PDAC)
Source: Cancers (Basel). 2023 Jan 19;15(3):628. doi: 10.3390/cancers15030628 (PMC9913151; doi:10.3390/cancers15030628)
Supplement: Supplementary file 1 [file cancers-15-00628-s001.zip › cancers-2127579-supplementary.pdf]

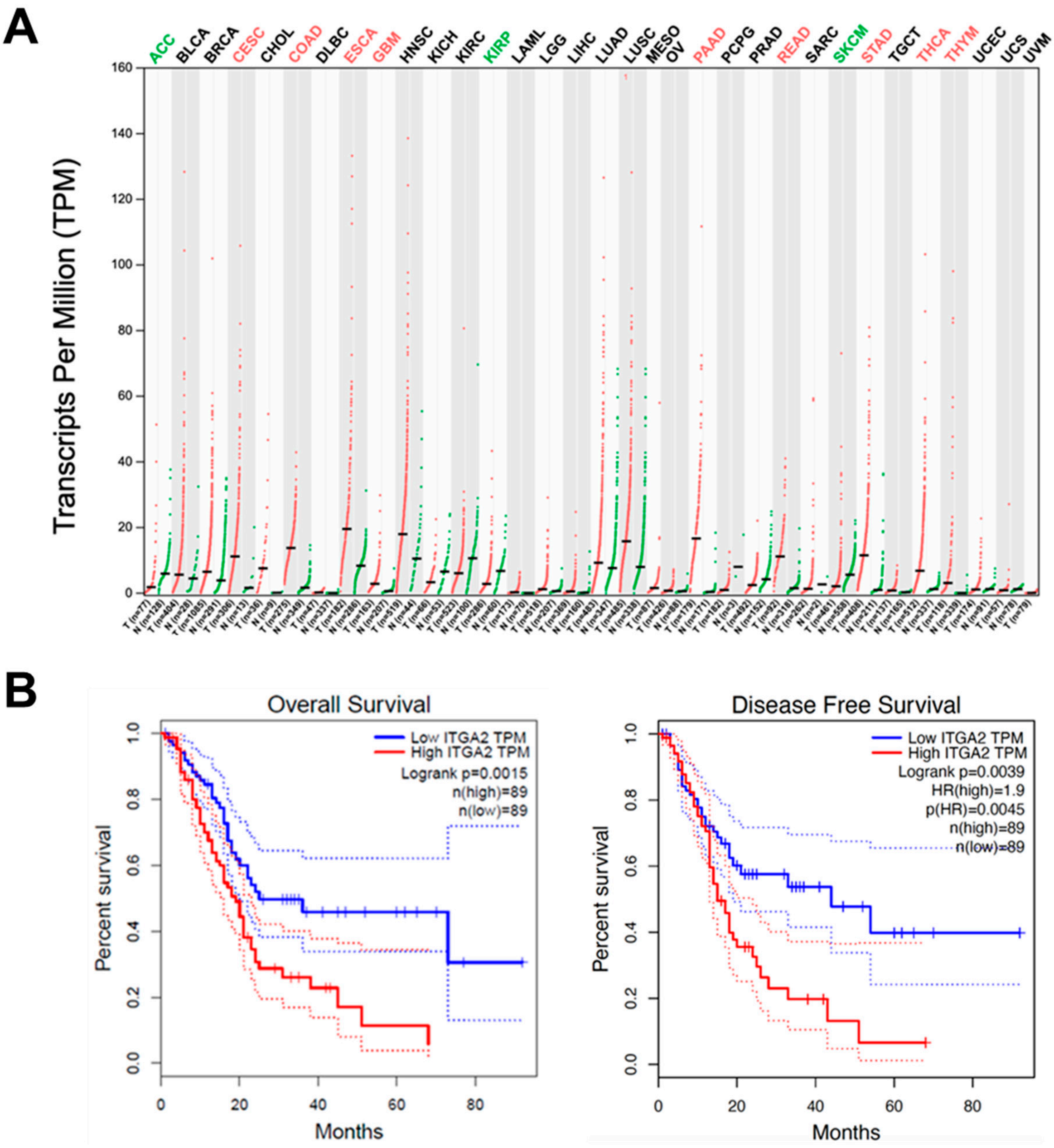

Supplemental Figure S1. ITGA2 expression profile and prognostic value in publicly available database. (**A**) ITGA2 expression profile across tumor samples and paired normal tissues. Each dots represent expression of samples. The square underlines the data of normal pancreatic tissues and patients affected by pancreatic ductal adenocarcinoma (PAAD). ITGA2 is more expressed in cancer tissues than in normal tissues and pancreatic cancer tissues are among the tumor tissues with the highest expression levels of ITGA2. (**B**) Evaluation of the ITGA2 prognostic value for PAAD patients from the GEPIA database. The patients were grouped according to the median value of ITGA2 mRNA expression and patients with low ITGA2 expression had a significantly longer overall survival and disease-free survival.

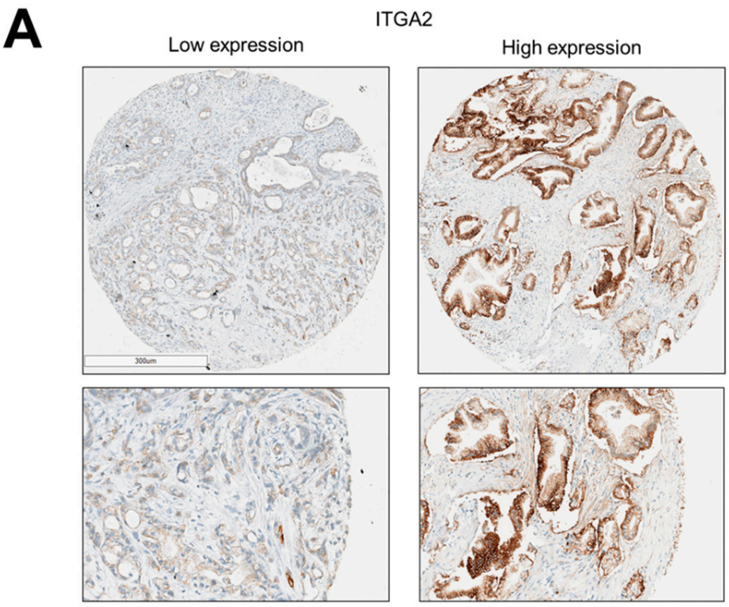

B

Scoring of ITGA2 protein expression staining					
	**Intensity of staining**		Weak	Middle	Strong
	Range	Score	1	2	3
**% Positive cells**	x=0	0	Low-ITGA2		
	0<x<25	1			
	25<x<75	2		High-ITGA2	
	x>75	3			

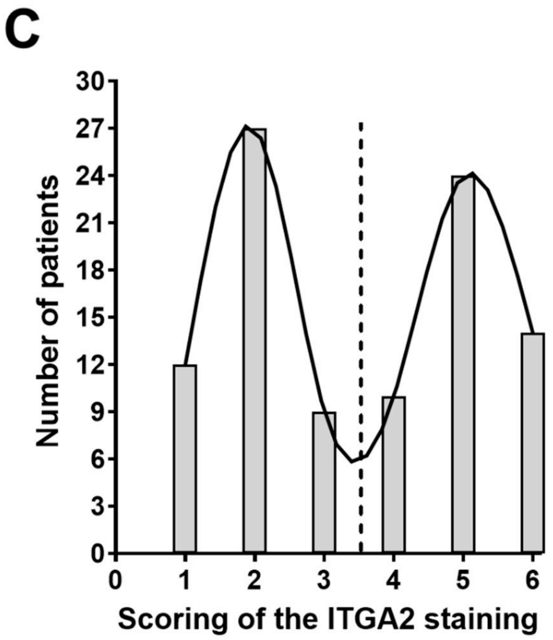

Supplemental Figure S2. Representative TMA cores and scoring system. (**A**) Representative TMA cores stained with ITGA2. Immunohistochemistry revealed a variable protein expression, related to the mRNA expression. Indeed, the tissues characterized by high *ITGA2* mRNA expression, presented a strong and diffuse staining of the ITGA2 protein (right panel), while the tissues with low *ITGA2* mRNA expression had only few scattered positive cells with a weak staining of the ITGA2 protein (left panel). Bottom panels show magnified areas of the respective cores. (**B**) Scoring of ITGA2 protein expression staining. The patients were grouped according to the median value of ITGA2 expression, which was 3.5. Thus the "high ITGA2" group included patients with tissues with the scores 4, 5 and 6, while the "low ITGA2" group included the scores 1, 2 and 3. (**C**) Distribution of the ITGA2 staining scoring among patients of the validation cohort.

A

	No, %	OS months (95% CI)	*P*	PFS months (95% CI)	*P*
No. Patients	45	10.7 (8.5-12.8)		7.2 (5.9-8.6)	
Age, y					
≤65	26 (58)	10.1 (7.7-12.5)	*0.37*	6.7 (5.1-8.2)	*0.25*
>65	19 (42)	11.3 (7.4-15.3)		8.0 (5.7-10.4)	
Sex					
Male	31 (69)	10.1 (7.3-12.8)	*0.52*	8.4 (5.4-11.3)	*0.16*
Female	14 (31)	12.0 (8.7-15.2)		6.7 (5.2-8.2)	
Grading					
1-2	21 (47)	13.4 (9.8-17.0)	***0.02***	8.6 (6.5-10.7)	***0.19***
3	24 (53)	8.2 (6.2-10.3)		6.0 (4.4-7.7)	
ITGA2 expression					
low	22 (49)	13.8 (10.7-16.9)	***0.01***	8.6 (6.8-10.8)	***0.03***
high	23 (51)	7.6 (5.3-10.0)		5.7 (4.1-7.4)	

B

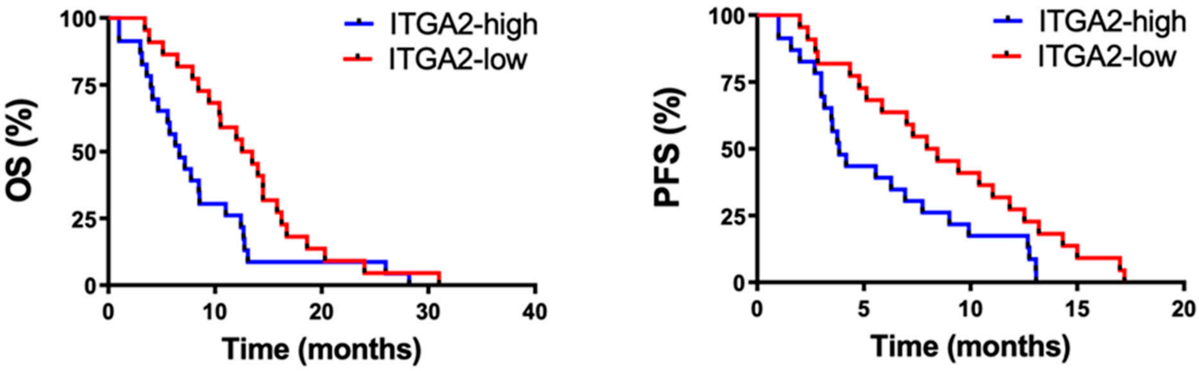

Supplemental Figure S3. Clinicopathological characteristics and Kaplan-Meier curves of the metastatic cohort. (**A**) Clinicopathological characteristics of the PDAC patients of the metastatic cohort. Abbreviations: PFS, Progression Free Survival; OS, Overall Survival; Significant P-values are in bold. (**B**) Kaplan-Meier curves of overall survival (OS, left panel) and progression-free survival (PFS, right panel).

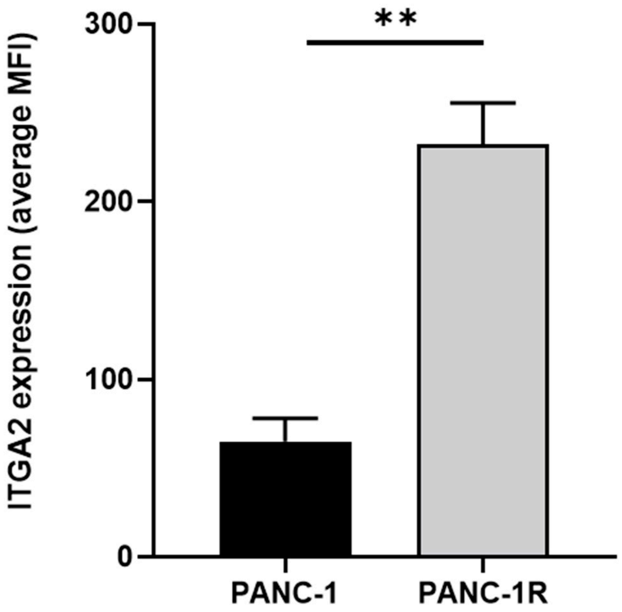

Supplemental Figure S4. Flow cytometry analysis of ITGA2 expression levels on the cell surface of PANC-1 and PANC-1R cells. Cells (1x10^6, technical triplicate analysis) were incubated in a blocking buffer (PBS with 1% foetal bovin serum) on ice for 20 minutes to inhibit unspecific binding of antibodies and then stained with the mouse anti-human ITGA2 monoclonal antibody (anti-ITGA2 Abcam, #ab115797, 1:100 dilution) at 4°C for 1 hour. The cells were then washed twice with PBS and incubated with a FITC-labeled goat anti-mouse IgG antibody (Abcam, #ab6785, 1:500 dilution) at 4°C for 30 minutes. As controls, we used human platelets (from peripheral blood), IgG control or only secondary antibody-stained samples. ITGA2 cell surface levels were measured with Fortessa (Beckton Dickinson (BD), Franklin Lakes, NJ) and results were analyzed with FlowJo™ v10.8 Software (FlowJo, LLC, BD), showing a 4-fold higher expression level of ITGA2 in PANC-1R (in black and grey, respectively) compared to PANC-1 cells, as detected by mean fluorescence intensity (MFI). Columns, mean values; bars, SD. ** $p<0.01$.

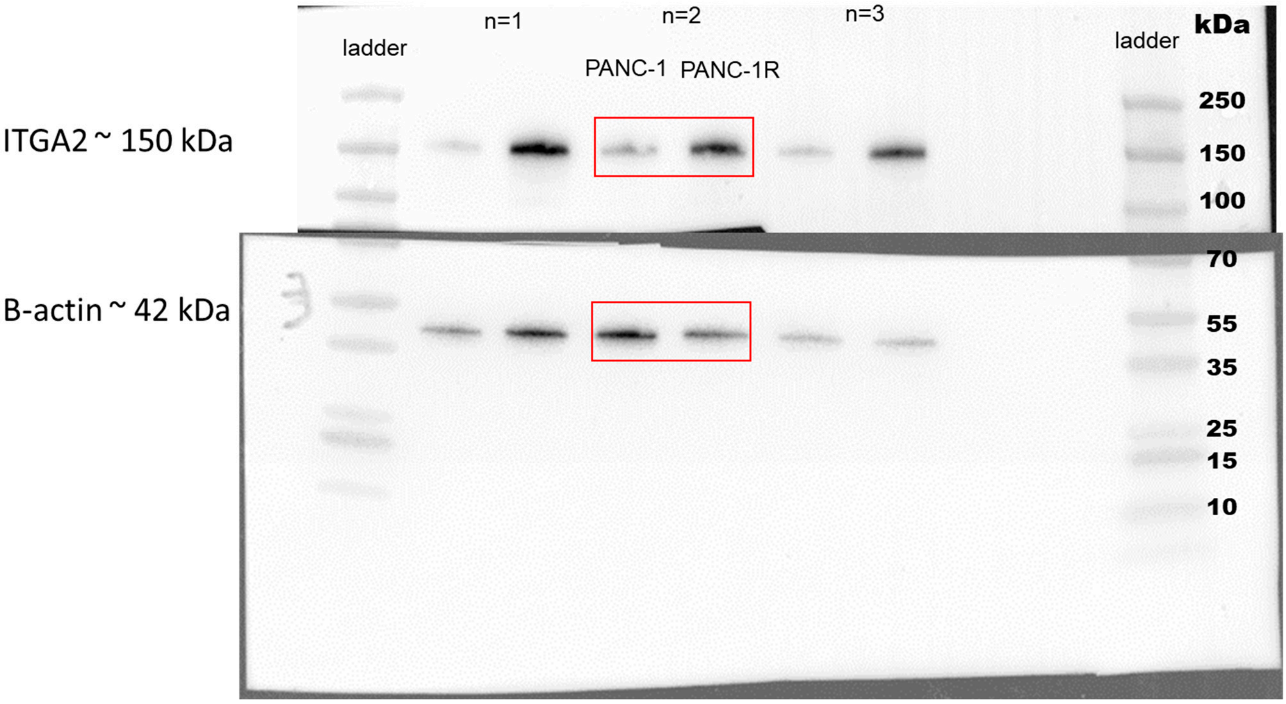

Supplemental Figure S5. Uncropped western blot membrane of PANC-1 and PANC1-R stained for ITGA2 and B-actin. Red boxes are the cropped areas used for representative image in figure 2C. Three biological replicates (n=1, 2 and 3) are displayed. Each sample was collected from untreated cells at least one week after each other to ensure PANC1-R were stably resistant.

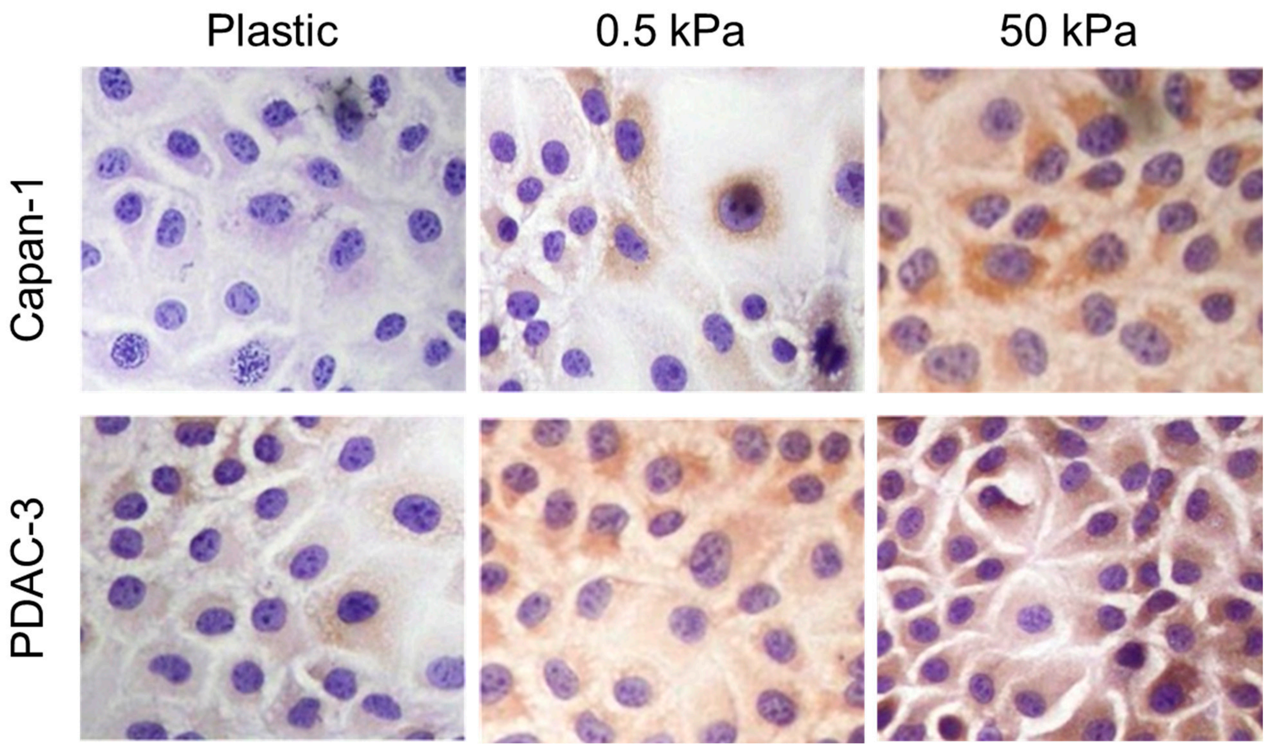

Supplemental Figure S6. Representative immunocytochemistry images of Capan-1 and PDAC-3 cell lines. ITGA2 expression level was analysed by immunocytochemistry for Capan-1 and PDAC-3 cells growing for 48 hours either on uncoated plastic or on soft and stiff collagen-coated bis-acrylamide gel (0.5 kPa and 50 kPa, respectively).

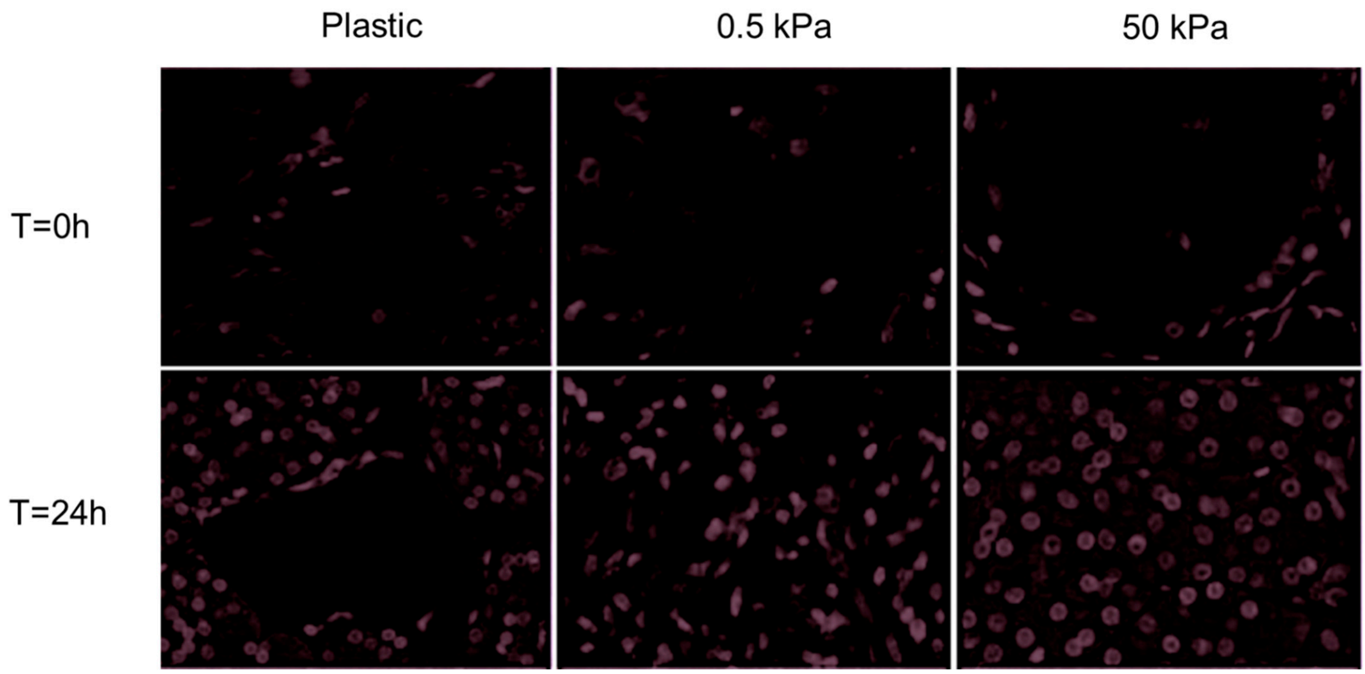

Supplemental Figure S7. Representative immunofluorescence images of the 2D wound healing assay. Cells were fixed using paraformaldehyde and then intracellular staining was performed in presence of triton x-100. Cells were then stained with propidium iodide, that binds to DNA thereby staining the nucleus, at 1:1000 concentration for 15 minutes.

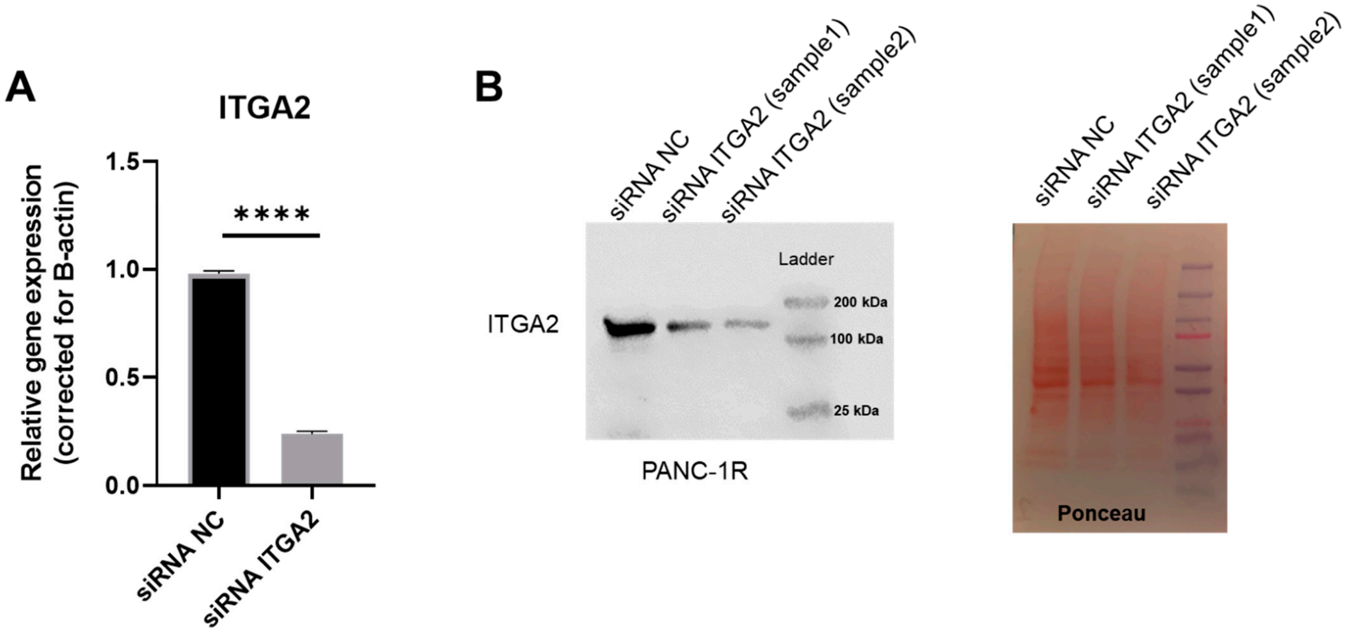

Supplemental Figure S8. ITGA2 knockdown efficiency in PANC-1R cells. (**A**) ITGA2 mRNA expression evaluated via qRT-PCR. Gene expression was normalized to house-keeping gene B-actin and calculated with the 2-ΔΔCt method. Data are expressed as mean ± SD of three experiments performed in duplicate. (**B**) ITGA2 protein expression evaluated by western blot. PANC-1R cells treated with either siRNA negative control (siRNA NC) or siRNA ITGA2 were stained for ITGA2 (left panel) and equal sample loading determined by Ponceau staining (right panel). Two biological replicates of siRNA ITGA2 (sample 1 and 2) are displayed.

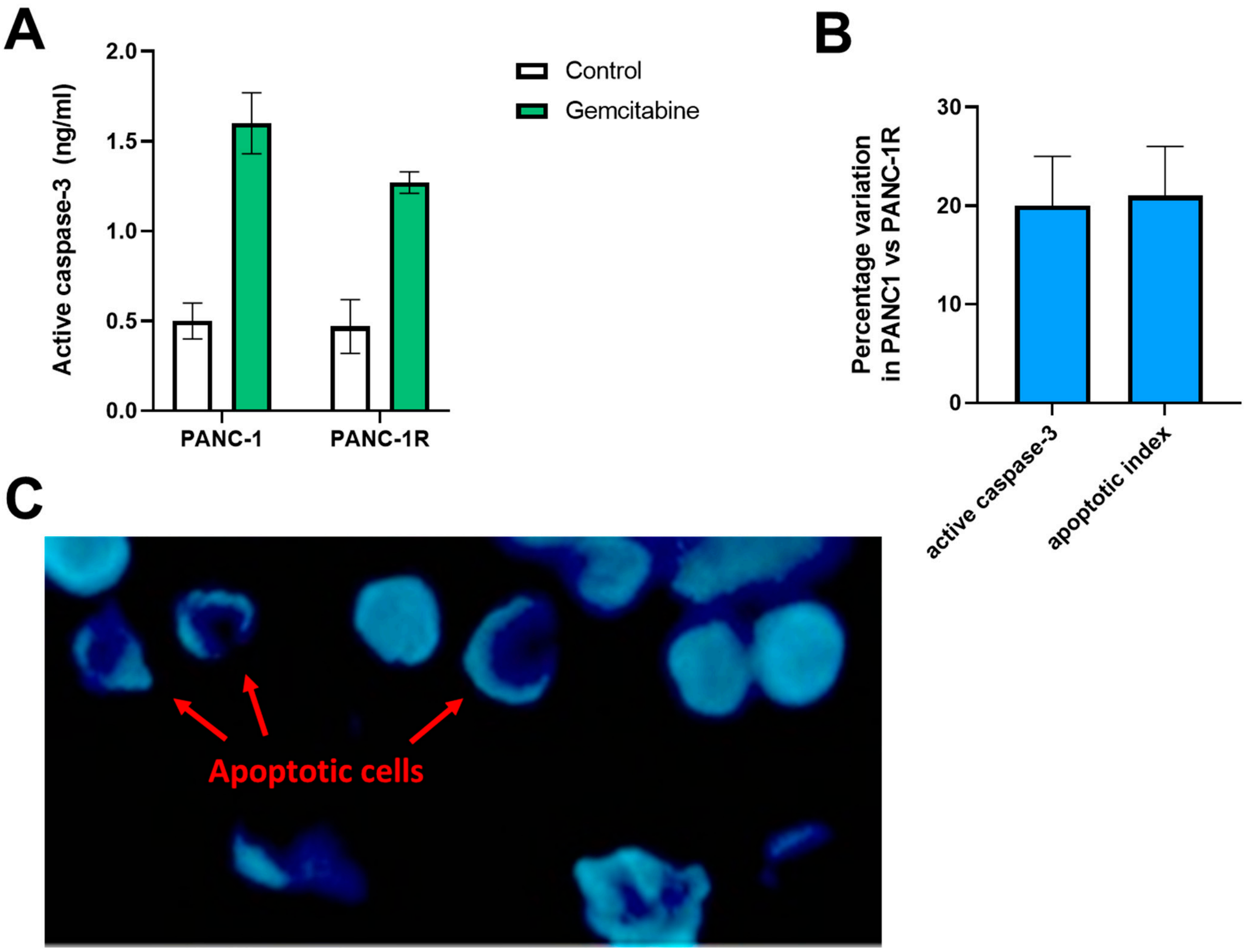

Supplemental Figure S9. Apoptosis induction in PANC-1 and PANC-1R cells. (**A**) Evaluation of apoptosis induction in untreated and cells treated with gemcitabine at IC_{50} by analysis of active caspase 3 (by Quantikine ELISA, R&D Systems, Inc., Minneapolis, MN). (**B**) Percentage of variation in apoptosis induction in PANC-1 vs. PANC1-R cells treated with gemcitabine IC_{50} using two different methods for the evaluation of apoptosis (active caspase-3 and bisbenzimide staining). (**C**) Representative image of PANC-1R apoptotic cells (red arrows) stained with bisbenzimide HCl solution, showing chromatin aggregation and nuclear fragmentation, which were evaluated to calculate the apoptotic index.

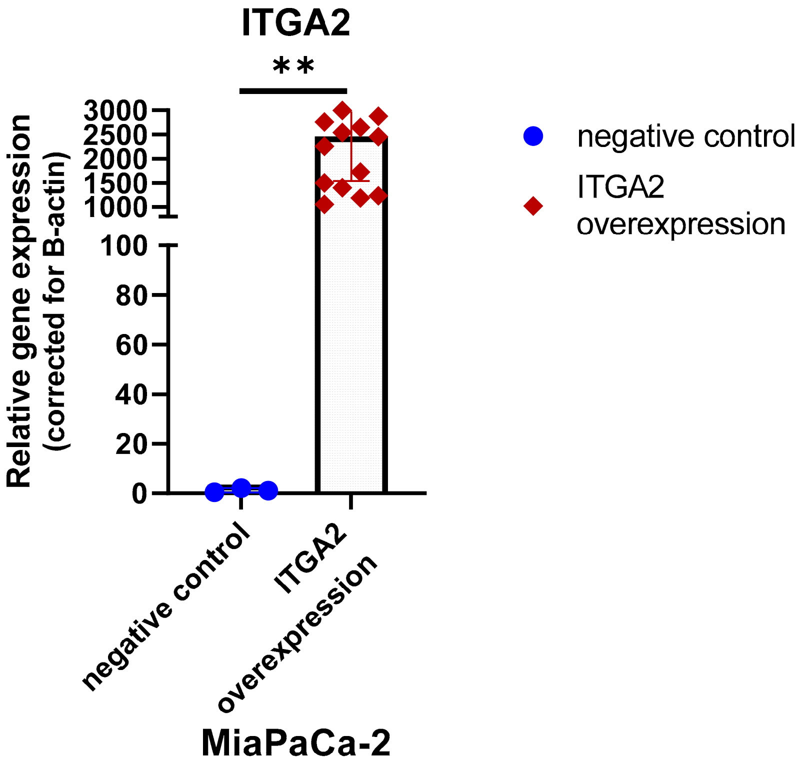

Supplemental Figure S10. ITGA2 overexpression in MiaPaCa-2 cells injected in mice. ITGA2 mRNA expression was evaluated in MiaPaCa-2 cells transfected with empty vector (blue = negative control) or with ITGA2-overexpressing vector (red = ITGA2 overexpression) and assessed by qRT-PCR. ITGA2 gene expression was calculated with the $2^{-\Delta\Delta Ct}$ method for three individual experiment performed in duplicate. ITGA2 was non-detectable in three out of six MiaPaCa-2 control samples, while the other three samples reported a Ct value close to the maximum threshold (39.5 cycles). Therefore, after adjustment to the housekeeping gene ΔΔCt was calculated using only the available Ct values as follows: ΔCt ITGA2 (n=6) – ΔCt control (n=3).
